# Supplementary material for: Once- versus twice-daily direct oral anticoagulants after ischemic stroke in atrial fibrillation – A post-hoc analysis of the ELAN trial
Source: Eur Stroke J. 2025 Aug 11:23969873251360974. Online ahead of print. doi: 10.1177/23969873251360974 (PMC12339485; doi:10.1177/23969873251360974)
Supplement: sj-docx-1-eso-10.1177_23969873251360974 – Supplemental material for Once- versus twice-daily direct oral anticoagulants after ischemic stroke in atrial fibrillation – A post-hoc analysis of the ELAN trial [file sj-docx-1-eso-10.1177_23969873251360974.docx]

**Supplementary Material**

**Once- versus twice-daily direct oral anticoagulants after ischemic stroke in atrial fibrillation**

**A post-hoc analysis of the ELAN trial**

**Supplementary Figure 1.** Study Flowchart

**384** included in the analysis of 90-day outcomes

**1506** included in the analysis of 90-day outcomes

**12** excluded

**4** withdrew consent

**0** lost to follow-up

**8** died from non-vascular causes

**56** excluded

**8** withdrew consent

**4** lost to follow-up

**44** died from non-vascular causes

**396** started on once-daily DOAC

**1562** started on twice-daily DOAC

**2013** comprising the full ELAN

analysis set

**1958** initiated DOAC and potentially eligible for this study

**55** excluded

**26** no DOAC initiation (missing DOAC type/initiation date)

**1** died from non-vascular causes

**1** experienced a study outcome event

**24** experienced other complications / other reasons

**29** experienced a study outcome before DOAC initiation

**Supplementary Figure 2.** Balance of baseline characteristics between participants with once-daily versus twice-daily DOAC assessed as standardized mean difference (SMD) before and after weighting. Weighting achieved good balance between the groups (absolute SMD < 0.05) across all baseline characteristics.


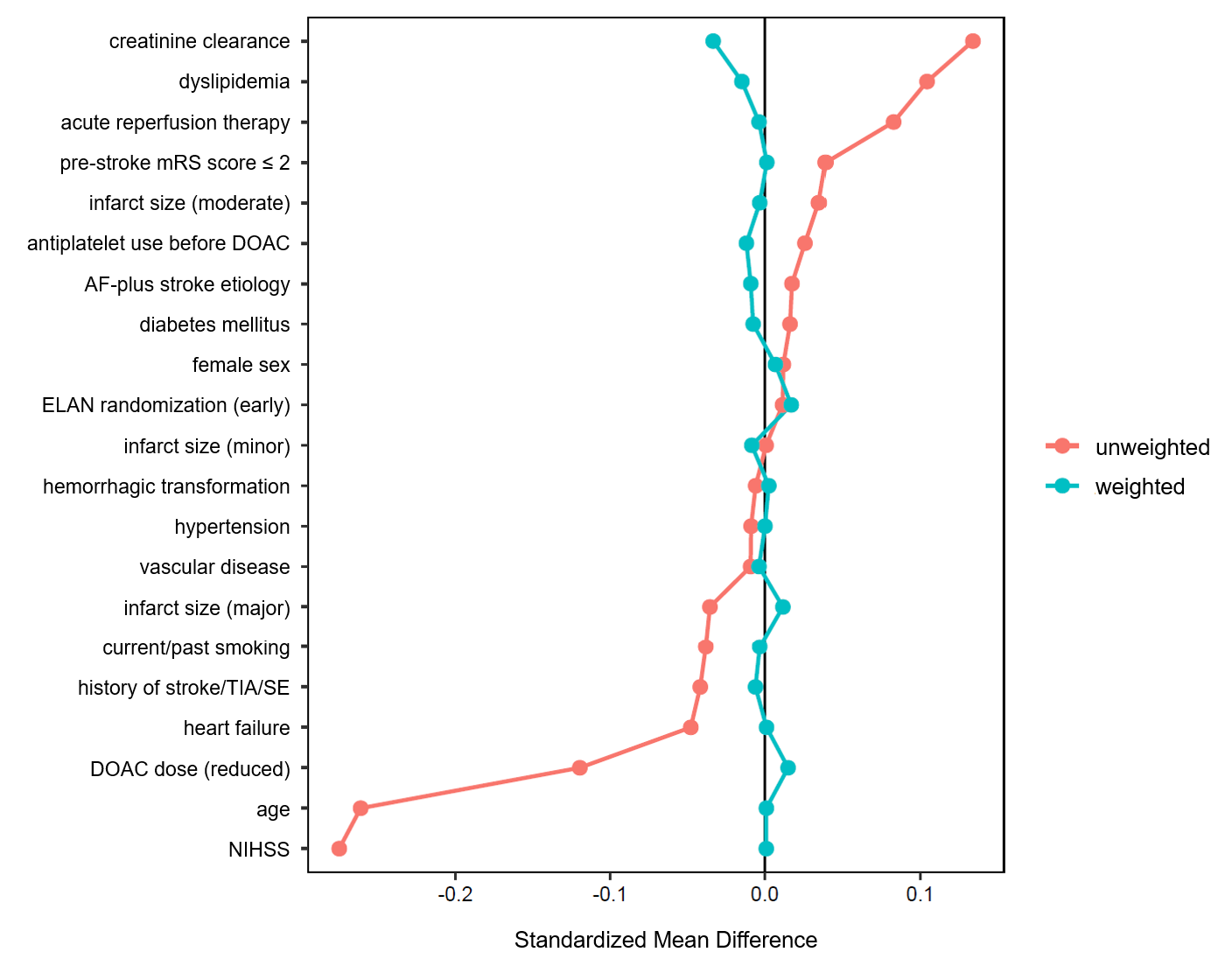


**Supplementary Figure 3.** Calculation of sample sizes needed to demonstrate a difference in the primary composite outcome given the treatment effect estimate from the logistic AIPW model (odds ratio of 1.45), a composite outcome rate of 3.6% with once-daily DOAC, and a ratio of once-daily to twice-daily DOAC of 1:3 (as in the ELAN trial) or 1:2 (as in the OPTIMAS trial) with a power ranging from 75% to 85% at an alpha of 0.05.

| **Supplementary Table 1.** Net clinical benefit of twice-daily versus once-daily DOAC | | | |
| --- | --- | --- | --- |
| **ICH weight** | **weighted rate of events* in participants with twice-daily DOAC (95%-CI)** | **weighted rate of events* in participants with once-daily DOAC (95%-CI)** | **Net clinical benefit of twice- versus once-daily DOAC (95%CI)** |
| 1.5 | 2.38 (2.78 to 5.68) | 2.66 (1.56 to 8.65) | +0.28 (-3.08 to +4.87) |
| 1.6 | 2.39 (2.87 to 5.78) | 2.69 (1.59 to 9.53) | +0.30 (-3.25 to +5.30) |
| 1.7 | 2.40 (2.82 to 5.94) | 2.72 (1.53 to 9.29) | +0.32 (-3.22 to +5.31) |
| 1.8 | 2.41 (2.85 to 5.76) | 2.76 (1.70 to 9.63) | +0.35 (-3.05 to +5.44) |
| 1.9 | 2.42 (2.84 to 5.81) | 2.79 (1.65 to 9.86) | +0.37 (-3.26 to +5.75) |
| 2.0 | 2.44 (2.92 to 5.87) | 2.82 (1.72 to 9.96) | +0.39 (-2.94 to +5.73) |
| 2.1 | 2.45 (2.85 to 5.91) | 2.86 (1.58 to 10.22) | +0.41 (-3.10 to +5.90) |
| 2.2 | 2.46 (2.93 to 5.93) | 2.89 (1.59 to 10.13) | +0.43 (-3.18 to +5.73) |
| 2.3 | 2.47 (2.86 to 5.85) | 2.93 (1.70 to 10.47) | +0.45 (-3.21 to +6.36) |
| 2.4 | 2.48 (2.99 to 6.05) | 2.96 (1.59 to 10.85) | +0.48 (-3.06 to +6.57) |
| 2.5 | 2.49 (2.90 to 5.97) | 2.99 (1.59 to 10.89) | +0.50 (-3.16 to +6.67) |
| 2.6 | 2.51 (2.98 to 6.26) | 3.03 (1.70 to 11.03) | +0.52 (-3.39 to +6.64) |
| 2.7 | 2.52 (2.80 to 6.16) | 3.06 (1.73 to 11.15) | +0.54 (-3.26 to +6.68) |
| 2.8 | 2.53 (2.88 to 6.16) | 3.09 (1.60 to 11.33) | +0.56 (-3.24 to +7.08) |
| 2.9 | 2.54 (3.02 to 6.28) | 3.13 (1.65 to 11.71) | +0.59 (-3.41 to +7.70) |
| 3.0 | 2.55 (2.94 to 6.21) | 3.16 (1.48 to 11.24) | +0.61 (-3.43 to +7.00) |
| 3.1 | 2.57 (3.07 to 6.11) | 3.20 (1.57 to 12.38) | +0.63 (-3.38 to +8.10) |
| 3.2 | 2.58 (2.98 to 6.24) | 3.23 (1.57 to 12.23) | +0.65 (-3.30 to +8.04) |
| 3.3 | 2.59 (3.02 to 6.47) | 3.26 (1.64 to 12.71) | +0.67 (-3.57 to +8.45) |
| *calculated as [rate of recurrent ischemic stroke + (0.9 x rate of systemic embolism) + (ICH weight x rate of ICH) + (0.7 x rate of major bleeding)]. All estimates are rates of weighted events per 100 participants. | | | |
